# Acupuncture for Erectile Dysfunction: A Systematic Review

**DOI:** 10.1155/2016/2171923

**Published:** 2016-01-17

**Authors:** Xiaoming Cui, Jing Zhou, Zongshi Qin, Zhishun Liu

**Affiliations:** ^1^Department of Acupuncture, Guang'anmen Hospital, China Academy of Chinese Medical Sciences, Beijing 100053, China; ^2^Beijing University of Chinese Medicine, Beijing 100029, China

## Abstract

*Background*. Acupuncture is increasingly used to treat patients with erectile dysfunction (ED), and our systematic review aimed to evaluate the current evidence for the efficacy and safety of acupuncture in treating ED.* Methods*. An electronic search was conducted in eight databases to identify randomized controlled trials (RCTs) of acupuncture for treating erectile dysfunction that were published in English and Chinese. The Cochrane Risk of Bias tool was used to assess the risk of bias.* Results*. Three RCTs with a total of 183 participants met the inclusion criteria. One trial showed the beneficial effects of acupuncture compared with sham acupuncture while the others did not. One trial suggested that acupuncture combined with psychological therapy was superior to psychological therapy alone. However, the overall methodological and reporting quality of the studies was low. The safety of acupuncture for ED was unclear because there were too few reports on this topic.* Conclusion*. The available evidence supporting that acupuncture alone improves ED was insufficient and the available studies failed to show the specific therapeutic effect of acupuncture. Future well-designed and rigorous RCTs with a large sample size are required. This trial is registered with CRD42014013575.

## 1. Introduction

Erectile dysfunction (ED), which is defined as the consistent inability to achieve or maintain an erection that is sufficient for satisfactory sexual performance, affects up to 52% of men between the ages of 40 and 70 years and is related to a reduced quality of life [1, 2]. It has been estimated that the prevalence of ED will be 322 million cases by 2025 worldwide [3]. There are various therapeutic options for treating ED, including psychosexual therapy, penile prostheses, revascularization, vacuum constriction devices, injection of vasoactive drugs, and oral drug therapy [4, 5]. However, these therapies are not always effective and may have adverse effects, such as penile pain, cardiovascular dysfunction, and deafness [5, 6]. Complementary and alternative medicine (CAM) is increasingly used to treat ED [7]. Acupuncture, which is defined as the insertion of needles into the skin and underlying tissues at acupoints for therapeutic or preventive purposes, is one of the most important components of CAM [8]. Contemporary research suggests that the neurophysiological influence of acupuncture, as a result of central nervous system activation and neurotransmitter modulation, may positively affect the pathophysiology of ED [9, 10]. As acupuncture is increasingly used in ED treatment, our systematic review aimed to evaluate the current evidence for the efficacy and safety of acupuncture for treating ED and to provide additional treatment options for ED patients.

## 2. Methods

A systematic review is reported here in accordance with the PRISMA guidelines [11] (S1 PRISMA Checklist). All screening, extraction, and assessment processes were conducted by two reviewers (Xiaoming Cui and Jing Zhou) who worked independently, and any disagreements were resolved by discussion with Zhishun Liu.

### 2.1. Protocol and Registration

A protocol of this systematic review was registered in the PROSPERO database (identification number: CRD42014013575) [12] and published in BMJ Open [13].

### 2.2. Inclusion and Exclusion Criteria

We determined the inclusion criteria according to the participants, interventions, comparator, and outcomes (PICOs) [14]; only randomized controlled trials (RCTs) were included. Study participants were required to be men aged ≥ 18 years with physical, psychological, or mixed ED diagnosed by any described criteria (e.g., DSM-IV [15], ICD-10 [16]). The intervention could be any type of acupuncture therapy except laser acupuncture and point injection. Studies comparing acupuncture (alone or combined with Chinese herbal medicine) and Chinese herbal medicine, two different forms of acupuncture, and different acupoints were excluded. The trials that lacked a validated questionnaire (e.g., International Index of Erectile Function (IIEF) [17]) or clear descriptions of the evaluation methods for outcomes were excluded.

### 2.3. Literature Search

The literature search was limited to articles published in English and Chinese that were included in the Cochrane Central Register of Controlled Trials (CENTRAL), Medline, PubMed, EMBASE, PsycINFO, Chinese Biomedical Literature Database (CBM), Chinese Medical Current Content (CMCC), and China National Knowledge Infrastructure (CNKI) from their inception to 20 March 2015. The following search terms were used individually or in combination: acupuncture, electro-acupuncture, fire needling, elongated needle, intradermal needling, dry needling, erectile dysfunction, impotence, and male sexual dysfunction. The specific search strategy was stated in the protocol of this review [13]. Grey literature, conference papers, and relevant references cited in selected studies were also searched as supplementary sources. The reviewers obtained and screened full texts of all remaining articles for eligibility.

### 2.4. Data Extraction and Quality Assessment

The data on the study methods and characteristics, participants, interventions, control, and outcomes were extracted from papers using a data extraction form. The authors were called or e-mailed to request provisions of any missing data. The reviewers assessed the risk of bias using the Cochrane Risk of Bias tool [18].

### 2.5. Data Analysis

Statistical analyses were conducted using Revman 5.3 software. To summarize the data, the relative risk (RR) and 95% confidence intervals (CI) were used for the effect size because the outcome of each included study was dichotomous. The heterogeneity of the included studies was assessed with Cochran's *Q* test and by examining *I* ^2^. Depending on the results, we chose a fixed or random effects model. However, meta-analysis was not performed because of the high statistical and clinical heterogeneity. As a result, we conducted systematic narrative synthesis that provides information to describe and explain the characteristics and findings of the included studies. A funnel plot was not used to assess publication bias because of the small number of included trials.

## 3. Results

### 3.1. Description of the Included Trials

**Figure 1:**
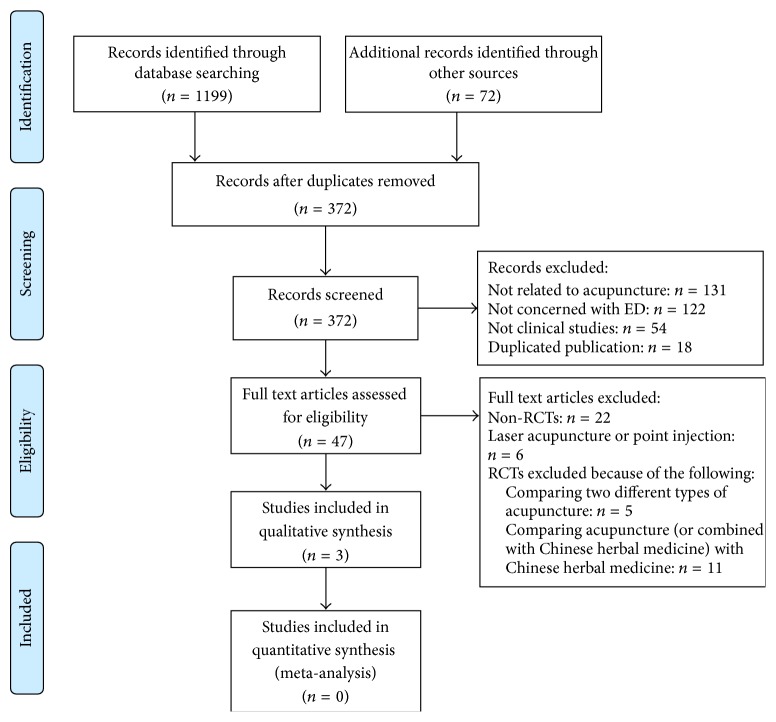
Flow chart of the study search. ED: erectile dysfunction; non-RCTs: non-randomized controlled trials; and RCTs: randomized controlled trials.

Figure 1 shows the PRISMA flowchart of the search and selection process. With a database search, we identified 1199 references; we reviewed 27 articles in the full text after screening the titles and abstracts. Finally, 3 RCTs met our inclusion criteria. Of the 3 trials, one was conducted in Turkey, one in Austria, and another in China. One [18] of the studies was a crossover design, while the others were parallel designs.

A total of 183 participants, with age ranging from 20 to 61 years, were involved and all of them were diagnosed with nonorganic ED.

With respect to the type of acupuncture, 1 study [19] investigated the effect of electroacupuncture (EA) and 2 studies [18, 20] investigated the effect of manual acupuncture (MA). The treatment duration varied from 12 to 30 sessions. Two studies [18, 19] compared acupuncture with placebo and one study [20] compared acupuncture plus psychological therapy with psychological therapy.

Two trials [19, 20] defined a positive treatment response as participants obtaining a sufficient erection for sexual intercourse, and one [18] defined effective treatment as treatment that made subjects “feel happy and (have) no problem with sexual activity.”

Aydin et al. [19] performed follow-up and reported that no adverse events occurred during acupuncture, and Engelhardt et al. [18] reported health economics. The participants' quality of life was not assessed in any of the studies. The details of the included trials are listed in Table 1.

### 3.2. Study Quality

**Figure 2:**
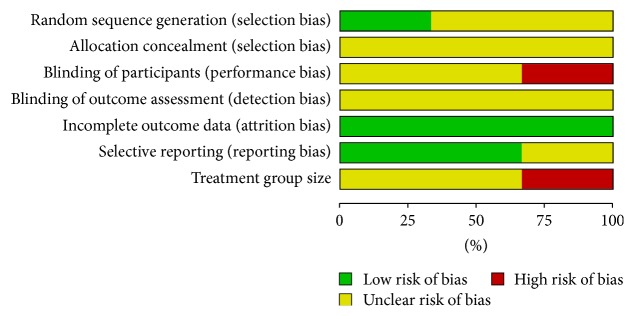
Risk of bias assessment. The percentages of studies with “low risk of bias,” “high risk of bias,” or “unclear risk of bias” are illustrated for each item of Cochrane Collaboration's tool. The assessment of risk of bias due to treatment group size: <50 participants per group indicated high risk of bias, 50–199 indicated unclear risk, and 200 or more indicated low risk.

**Figure 3:**
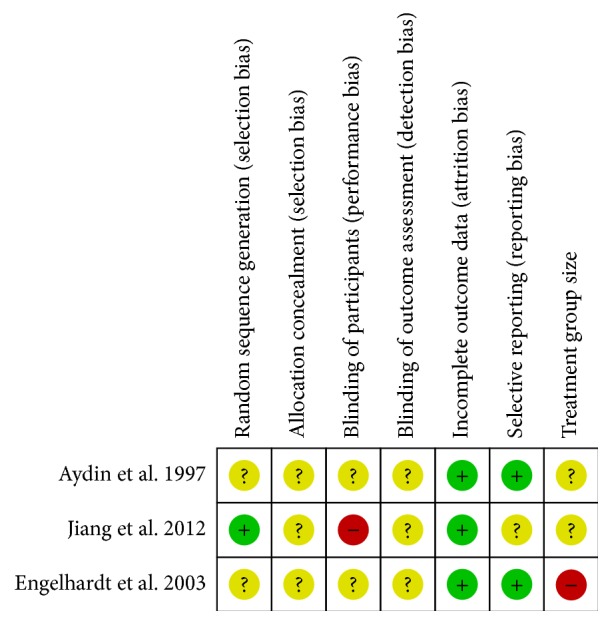
“Risk of bias” summary: review of the authors' judgments about each risk of bias item for each included study.

Results of the risk of bias assessment are presented in Figures 2 and 3. All studies mentioned “random”, but only one [20] of them described the randomization method as a random number table. The process of allocation concealment and blinding was unclear in all of studies even though we tried to gain more details by contacting the authors.

The performance bias of one trial [20] was judged as “high” because the comparis**o**n groups did not allow for blinding of the participants and personnel. One trial [18] reported the dropout rate for personal reasons without performing intent-to-treat (ITT) analysis. The overall quality of reporting was low, as reflected by the large proportions of “unclear risk of bias.”

### 3.3. Effect Estimates

#### 3.3.1. Acupuncture versus Sham Acupuncture

Two trials compared the effects of acupuncture with sham acupuncture.

Engelhardt et al. [18] investigated the effect of MA for an average of 11 treatment sessions on 21 patients who suffered from psychogenic ED for an average of 23.8 months by interview and IIEF questionnaire. The treatment group underwent acupuncture that was performed at the traditional acupoints for ED, whereas the sham group was treated with acupuncture that was performed at points for a headache. Acupuncture was considered effective when the erection became sufficient for sexual intercourse. After 4–10 sessions, 10 of 11 patients in the sham group did not show improvement and crossed over to the treatment group. Overall, after acupuncture treatment, 13 of 19 patients (including 10 patients after crossover) could obtain a full erection without further therapy, and their mean IIEF score improved by 41.9%. MA showed significant effects on improving erectile function compared with the sham group (RR 7.53, 95% CI: 1.13–50.00, *P* = 0.04).

Aydin et al. [19] tested the effect of EA for 12 treatment sessions on the improvement in sexual activity by interviewing 60 patients with nonorganic ED. The treatment group received EA with needles inserted into traditional acupoints on the legs and abdomen. In the sham group, EA was punctured into nonpoint, nonmeridian sites on the legs and arms. Patients were reported as “cured” if “they felt happy and had no problem with sexual activity” and their partners independently verified the report. After six weekly treatments, 60% of the EA group was “cured” compared with 43% in the placebo group. However, EA did not have significant superiority (RR 1.40, 95% CI: 0.67–2.91, *P* = 0.37).

The studies' statistical and clinical heterogeneity prevented us from conducting meta-analyses even though we had originally intended to perform a meta-analysis (*χ* ^2^ = 3.56, *P* = 0.06; *I* ^2^ = 72%).

#### 3.3.2. Acupuncture plus Psychological Therapy versus Psychological Therapy

Jiang et al. [20] tested the effectiveness of MA plus psychological therapy compared with psychological therapy alone on 102 patients who had psychogenic ED for an average of 3.65 years. The control group received psychological therapy 24 times in a month that was aimed to help patients improve their confidence. The treatment group received the same psychological therapy as the control group. At the same time, they were treated with acupuncture once a day. The treatment was reported as “effective” if the patients could achieve a full erection for intercourse. After 30 sessions, treatment was “effective” for 45 of 51 patients in the treatment group and 24 of 51 patients in the control group. Overall, there was a significant difference between the treatment and control groups (RR 1.88, 95% CI: 1.38–2.55, *P* < 0.0001).

## 4. Discussion

### 4.1. Summary of the Main Results

This systematic review summarizes the available evidence from randomized controlled clinical trials for the efficacy of acupuncture on ED. Three RCTs that met our inclusion criteria were performed on patients with nonorganic ED although we tried to find more evidence for both physical and psychological ED.

The limited evidence for the two RCTs comparing acupuncture with sham acupuncture failed to show a specific therapeutic effect of acupuncture, which was also supported by the previous review [21].

Engelhardt et al. [18] reported favorable results, while Aydin et al. [19] did not. Moreover, both studies had methodological problems, such as an unclear randomization and allocation concealment process, small sample sizes, and a lack of assessor blinding. Neither of the studies assessed the success of blinding, although they adopted a sham control. Engelhardt et al. [18] used MA insertion into acupoints for headache for sham control, while Aydin et al. [19] used EA insertion into points other than classical acupoints for sham control. We did not combine these two studies because there was high heterogeneity. The significant heterogeneity might result from the different types of trials; one had a partial crossover design and the other was parallel.

Although acupuncture was not significantly superior to sham acupuncture, the 60% improvement with EA reported by Aydin et al. [19] and 68.4% improvement with MA by Engelhardt et al. [18] might suggest that acupuncture could be an alternative adjuvant treatment for psychological ED. Acupuncture may affect the spinal sensorial level and have a central effect because the patients could acquire “peace of mind” [22]. Lowering the anxiety and reducing the mental stress might help improve psychological ED. It is possible that acupuncture could act as a strong placebo, and it might also be meaningful as an alternative adjuvant treatment for psychological ED. Moreover, the results of Jiang et al. [20] supported this concept. The effect of acupuncture combined with psychological therapy was much better than psychological therapy alone. Acupuncture might be an add-on therapy for psychological therapy in the treatment of psychological ED, although the evidence was not sufficiently powerful to draw a firm conclusion because of the small simple size and unclear risk of bias.

There were no adverse events in one trial, while the others did not assess or mention adverse effects. Although acupuncture is commonly considered safe, assessing and reporting adverse effects are necessary because numerous adverse events have been reported [23]. As a result, the safety of acupuncture for ED is uncertain because of the inadequate reporting of adverse events.

### 4.2. Limitations

The small number of studies included in this report limits our findings. The small sample size and poor quality of the trials might cause bias. The review only included studies that were published in English and Chinese, and it is possible that some studies in other languages were not found. However, a large number of different databases were searched, and the review was strictly conducted according to our published protocol.

### 4.3. Future Perspectives

More high-quality RCTs on the use of acupuncture to treat ED with larger sample sizes are expected. The use of a placebo, standard Western medicine, or therapy for ED as a control is also needed to assess the clinical value of acupuncture. Moreover, we propose that a generally acknowledged questionnaire or scale, such as IIEF-5, should be preferably adopted as the outcome measures of future trials. Adverse events should be identified and reported in full.

## 5. Conclusion

The available evidence for the ability of acupuncture alone to improve ED was insufficient and previous studies have failed to show the specific therapeutic effect of acupuncture for treating ED. Future well-designed and rigorous RCTs with a large sample size are needed.

## Figures and Tables

**Table 1:** Summary of the included studies.

Author, year, country	Design, sample size/conditions	Experimental intervention	Control intervention	Primary outcome measures	Acupuncture points
Aydin et al., 1997, Austria [19]	RCT, 60 patients with nonorganic ED (mean age 36.7 y)	EA (3 Hz dc, 20 min, twice a week for 6 weeks) *n* = 15	(1) Hypnotic suggestions (three times a week, later once a month) *n* = 16 (2) Oral placebo (vitamin pill) *n* = 15 (3) Placebo needle (3 Hz dc, 20 min, twice a week for 6 weeks) *n* = 14	Improvement in sexual activity (interview)	True: Qichong (ST30), Zusanli (ST36), Zhaohai (KI6), Guanyuan (CV4), Qihai (CV6) Sham: different points compared to classical acupuncture points

Engelhardt et al., 2003, Austria [18]	RCT, 21 patients with psychogenic ED (mean age 38.9 y)	MA (20 min, once or twice a week, 5–20 sessions, mean 11) *n* = 10	Sham MA (20 min, once or twice a week, 4–10 sessions, mean 6.2) *n* = 11	(1) Response rate (interview) erection sufficient for penetration and intercourse (2) IIEF-5 score	True: Zhaohai (KI6), Shufu (KI27), Guanyuan (CV4), Qihai (CV6), Wangu (SI4), Sanyinjiao (SP6), Shenshu (BL23) Sham: Xuanzhong (GB39), Jiexi (ST41), Tianshu (ST25) (3 acupoints for headache)

Jiang et al., 2012, China [20]	RCT, 102 patients with psychogenic ED (mean age 28.7 y)	MA (30 min, once a day, 30 days) plus psychological therapy (5–15 min, 12 times/15 days) *n* = 51	Psychological therapy (5–15 min, 12 times/15 days) *n* = 51	Response rate (interview) erection sufficient for penetration and intercourse	Qihai (CV6), Guanyuan (CV4), Zhongji (CV3), Sanyinjiao (SP6), Taichong (LR3), Xingjian (LR2), Taixi (KI3), Yongquan (KI1), Neiguan (PC6), Shenmen (HT7), Baihui (GV20), Xinshu (BL15), Shenshu (BL23), Qihaishu (BL24), Mingmen (GV4)

ED: erectile dysfunction; RCT: randomized controlled trial; EA: electroacupuncture; MA: manual acupuncture; IIEF-5: International Index of Erectile Function-5. Acupuncture points designated according to WHO guideline.
